# Invasiveness of endometrial cancer cell lines is potentiated by estradiol and blocked by a traditional medicine Guizhi Fuling at clinically relevant doses

**DOI:** 10.3389/fonc.2022.1015708

**Published:** 2023-01-16

**Authors:** Sidra Khan, Alanah Varricchio, Carmela Ricciardelli, Andrea J. Yool

**Affiliations:** ^1^School of Biomedicine, University of Adelaide, Adelaide, SA, Australia; ^2^Adelaide Medical School, Robinson Research Institute, University of Adelaide, Adelaide, SA, Australia

**Keywords:** endometrial cancer, cell invasiveness, guizhi fuling, phytomedicine, women’s reproductive health, estradiol, progestin

## Abstract

The Traditional Chinese medicine, Guizhi Fuling (here called Fuling), has been confirmed in meta-analysis studies to reduce recurrence of endometriosis and improve pregnancy outcomes; however, the possible use of Fuling as a fertility-preserving treatment in endometrial cancer has not previously been tested. Results here are the first to demonstrate dose-dependent inhibition of cell motility by Fuling in two endometrial cancer cell lines, classified as Grade I which is responsive to progesterone treatment, and Grade III (MFE-280) which is resistant. The major outcome of this study was the novel demonstration that Fuling (30-80 µg/ml) significantly inhibits invasiveness in both high and low grades of EC cells, achieving 70-80% block of trans-barrier migration without cytotoxicity. This effective dose range is estimated to be comparable to that used in human clinical trials and traditional practice. Results here further show that clinically relevant doses of Fuling override the motility-promoting effects of estradiol in endometrial cancer cell lines. Medroxyprogesterone acetate has to date been the standard therapy to treat metastatic or inoperable endometrial cancers; however, success rates are low with high rates of recurrence, due in part to acquired resistance to medroxyprogesterone acetate therapy. The discovery here that Fuling appears to control the spread of treatment-resistant advanced cancers is an exciting prospect.

## 1 Introduction

Endometrial cancer (EC) arises from over-proliferation and spreading of cells from the wall of the uterus, constituting the most common gynecological malignancy worldwide and the sixth most common cancer among females, affecting approximately 17 per 100,000 women (1, 2). These cancers arise from endometrial gland cells and account for 75-80% of uterine cancer cases (3, 4). Endometrial cancers have traditionally been classified into Types I (endometroid) and II (non-endometroid) according to clinical, pathological and molecular features, as well as patient prognoses (3, 5). Type I endometroid tumors have been linked to unopposed estrogen action, high body mass index (BMI), hormone receptor-positivity and endometrial hyperplasia, and associated with more favorable prognoses. In contrast, Type II endometrial tumors have been linked with low or normal BMI, negative hormone receptor status, endometrial atrophy, and poorer prognoses (5). The Bokhman classification scheme has been useful for EC research and generally remains valid for low grade endometroid cancers; however, in high grade endometroid and serous carcinomas, overlapping histological features elude this traditional classification scheme (6). Current views acknowledge that EC comprises a heterogeneous set of diseases with diverse morphological, clinical, and genetic features.

Genomic analyses of endometroid, serous, and mixed endometroid and serous cancers compiled by the Cancer Genomic Atlas Research Network (TCGA) in 2013 (7) support categorization of EC into four sub-types: Class 1: POLE (Polymerase Epsilon exonuclease domain mutated tumors which are characterized by high mutation rates in *PTEN*, *PIK3R1*, *PIK3CA*, *FBXW7* and *KRAS* genes, and associated with more favorable prognoses; Class 2: MSI [Microsatellite-instable (hypermutated)] which comprise low grade endometroid tumors showing high mutation rates, microsatellite instability, and *KRAS* and *PTEN* mutations; Class 3: CNL (copy-number low) tumors also known as the ‘endometroid sub-group’ which are low grade tumors with low mutation rates and frequent *CTNNB1* mutations; Class 4: CNH (copy-number high) tumors also referred to as the ‘serous sub-group’ which include a combination of serous, mixed, as well as some grade 3 endometroid tumors, characterized by recurrent *TP53* mutations and poor outcomes. A meta-analysis study by Travaglino et al. (8) indicated that FIGO (Fédération Internationale de Gynécologie et d’Obstétrique) categories defined as grades I and II (with less than 50% differentiated glandular tissue) fell into the CNL and MSI groups with favorable outcomes, whereas FIGO grade III (with more than 50% glandular tissue and poor differentiation) fell into POLE and CNH groups with poor prognoses.

Our study utilized two endometrial cancer cell lines: *(i)* Ishikawa with properties of FIGO grade I (low grade) endometroid cancer cells; and *(ii)* MFE-280 with properties of FIGO grade III (high grade) endometroid cancer cells. The Ishikawa cell line originally was established from an endometroid cancer from a 39-year-old pre-menopausal woman, and has been shown to express estrogen and progesterone receptors (9). According to the Expasy Cellosaurus database, the Ishikawa cell line (CVCL_D199) carries *PTEN* mutations, consistent with TGCA-based classification as MSI. The MFE-280 cell line was derived from a recurrent, poorly differentiated endometrial carcinoma from a 78-year-old post-menopausal patient (10). Based on Expasy Cellosaurus data, the MFE-280 cell line (CVCL_1405) carries *TP53* gene mutations, consistent with the TGCA classification as CNH.

Pathological features of endometroid cancer are associated with conditions that promote estrogen predominance unopposed by anti-proliferative actions of progesterone, including for example cases of polycystic ovarian syndrome, nulliparity, hormone replacement therapy, obesity, hypertension and glucose intolerance (metabolic syndrome) (3), all potentially acting to augment hypertrophy of the endometrium (11). Progesterone deficiency has been proposed as a critical component of the increased risk of endometroid cancer for patients with associated risk factors of estrogen replacement therapy or obesity, with obesity being more closely linked with risk of low grade than high grade tumors (12, 13). Endometroid cancer is usually diagnosed at an early stage by distinctive symptoms of postmenopausal bleeding (14). Outcomes are improved if treatment is started promptly (3, 15), but the effectiveness of available treatment options remains limited. The first-line therapy is surgery (removal of uterus and both ovaries) (15) although this does not preclude recurrence, and the compromised fertility and childbearing ability along with symptoms of menopause due to removal of ovaries can exacerbate psychological and sexual distress (16). Alternatives intended to be fertility sparing using chemotherapy, radiotherapy, and progestin therapy would benefit from more clinical trial data establishing long-term benefits for pregnancy-related outcomes. Although synthetic progesterone can be effective particularly for early stage and low-grade cancers (17), a substantial proportion of patients fail to achieve complete responses and show cancer recurrence (18, 19). Combined therapies (such as radio- and chemotherapies) can restrict regional spread within the organ but still fail to control wider metastasis or improve overall survival, especially in patients with high grade tumors and extensive myometrial and lymph vascular invasion (15). Non-surgical and nontoxic fertility sparing treatment options are needed.

One promising candidate for pharmaceutical intervention is the Traditional Chinese Medicine, Guizhi Fuling (here referred to as Fuling), an herbal extract with medicinal properties first noted in the third century by Han Dynasty physician Zhang Zhongjing who used Fuling for treatment of dysmenorrhea (20). Fuling has been explored as a treatment for endometriosis, chronic inflammatory disorders, brain ischemia, reperfusion injury, chronic dysmenorrhea, and uterine fibroids (21, 22). Anti-tumor effects of Fuling have been identified in cancers including bladder (23), hepatocellular (24), and cervical (21). Our analysis of published literature identified a limited number of studies that have tested the effects of Fuling and candidate signaling pathways in animal and cellular models of reproductive disorders; these are summarized in **Table 1**.

**Table 1:** Summary of published work investigating the effects of Fuling in *in vitro* and *in vivo* models of reproductive system pathologies, with proposed candidate signaling pathways.

Pathology	Model	Dose	Outcome	Candidate pathway	Ref
Ovarian cancer	HEY-T30, SKOV3 cell lines (Papillary cyst-adeno-carcinoma; Ovary adeno-carcinoma) Mouse model (BALB/c female) of ovarian cancer	3 mg/ml 30 mg/kg intragastric gavage	Inhibition of colony formation; Inhibition of invasion Increased apoptosis in tumor cells	Regulates galactose and fatty acid metabolism in mitochondria	(25)
	HEY-T30 and SKOV3 cell lines Rat model of ovarian cancer	1.5-24 mg/ml 30 mg/kg intragastric gavage	Decreased cell viability, inhibition of invasion, increased apoptosis Increased apoptosis in tumor cells	Inhibits TGF-β1 induced migration and invasion. Downregulates expression of AKT/GSK3β signaling pathways	(26)
	SKOV3/DDPcell lines	4000-16000 mg/kg from rat sera	Restored sensitivity of cell line to cisplatin.	Upregulates mRNA and protein expression of tumor suppressor gene PTEN	(27)
	HEY-T30, SKOV3 cell lines	0.75-24 mg/ml	Inhibition of migration at 3mg/ml	Downregulates PHD finger protein 19	(28)
Cervical cancer	HeLa cells	0.005 and 0.01 mg/ml	Inhibition of transwell invasion	Downregulates matrix metalloproteinases MMP-2 and MMP-9; and upregulates tissue inhibitor of MMP (TIMP)	
Endometriosis	Rat model of endometriosis (female Sprague Dawley)	4130, 8260, 16250 g/kg intragastric gavage	Increased apoptosis of uterine endothelial cells	Downregulates monocytic chemoattractant protein (MCP-1), cell adhesion molecule (ICAM -1). Activates CD4+ and natural killer (NK) cells. Upregulates apoptotic genes caspase-3, caspase-9. Downregulates inhibitor gene of apoptosis, surviving, bcl-2. Upregulates pro-apoptotic gene Bax.	(29, 30)
Endometriosis	Rat model of endometriosis (female Sprague Dawley)	1912 mg/kg, 480 mg/kg intragastric gavage	Decreased endometriotic implant size	Increases CD4+ and NK cells Decreases mRNA and protein expression of MCP-1 and ICAM-1	(30, 31)

Prior work has shown treatments with high doses of Fuling cause cell death (**Table 1**), with concomitant reductions in invasiveness that could be due indirectly to the loss of cell viability. High doses of Fuling in a mouse model of ovarian cancer suppressed tumor growth and distant metastases, and reduced *in vitro* proliferation and invasiveness of ovarian cancer cells (25, 28). In breast cancer, the ability of Fuling to inhibit cell invasion is not known; however, high throughput screening (HTS^2^) assays of gene expression after exposure to Fuling suggested possible involvement of PI3/Akt (29), an actin remodeling pathway involved in cell motility. Cervical cancer HeLA cells treated with low doses of Fuling showed inhibition of cell death, as summarized in **Table 1**. Overlooked roles of low doses of Fuling on processes such as restricting invasive cell spreading and metastasis merit further exploration.

Beneficial effects of Fuling in endometriosis have been supported by clinical studies in humans. A decreased risk of recurrent endometriosis after Fuling treatment was confirmed in a meta-analysis of ten randomized human clinical trials, in which Fuling was administered in combination with the standard anti-progesterone therapy mifepristone (RU486) (32). The treatment combination not only lowered the risk of adverse effects associated with RU486 (irregular vaginal bleeding, hot flushes, abnormal liver function), but also improved pregnancy outcomes (32). Fuling is currently being investigated under the Registry of Australia New Zealand Clinical Trials (ACTRN12619000807156) as “A Clinical Trial to Test a Modified Traditional Chinese Herbal Medicine for the Treatment of Endometriosis” (33, 34). In this trial, patients with endometriosis are receiving six Fuling (Gynoclear) capsules (3.3 g per day) orally for 12 weeks. While acknowledging that Fuling components and their drug disposition and metabolism remain to be defined, we can nonetheless as a starting point estimate that for an average adult woman (~50 liters of body fluid), a systemic intake of 40 mg/kg could be roughly estimated as an effective dose of about 70 µg/ml per day. While not intended to be definitive, this estimate provides a useful starting point for framing doses to be tested in experimental work investigating biological mechanisms of actions of Fuling.

Endometriosis and endometrial cancer share common features in terms of molecular pathogenesis, overgrowth, and resistance to apoptosis (35–37). Surprisingly however, the effects of Fuling on endometrial cancer have not previously been assessed. Work here evaluated treatments with a broad range of Fuling doses in two grades of EC cell lines. The low Fuling doses used here are estimated to be relevant to those used in *in vivo* human clinical studies of endometriosis; the high doses are equivalent to those shown in prior work to kill a variety of reproductive cancer cell types. Results here are the first to show that Fuling at low non-cytotoxic doses is highly effective as an inhibitor of invasiveness in high- and low-grade endometrial cancer cells. These effects are consistent with the suggested clinical benefits of Fuling in patients with endometriosis.

Fuling shows promise as a novel adjunct for the clinical management of both low grade and high grade endometrial cancers, but confirmation of this idea awaits efficacy testing *in vivo*.

## 2 Materials and methods

### 2.1 Chemicals and reagents

Dulbecco’s Modified Eagle’s Medium (DMEM, #12430), fetal bovine serum (FBS), GlutaMAX™ (#35050061), penicillin-streptomycin (#15070-063), phenol red-free DMEM/F12 (#21041025) were purchased from Merck (Sigma-Aldrich; Sydney NSW, Australia). 17β-estradiol (E8875-250MG; referred to here as estradiol), synthetic progesterone (P0130-25G), estrogen receptor antagonist, ICI182,780 (129453–61–8), and the progesterone receptor antagonist mifepristone, referred to here as RU486 (M8046) were obtained from Merck. Insulin from bovine pancreas (I6634-25mg) also was from Merck.

### 2.2 Cell culture

Ishikawa 3-H-12-Luc (JCRB1579, Ishikawa, purchased Jan 2019 at passage 4) and MFE-280 (ECACC 98050131, purchased Jan 2021 at passage 68) cell lines were obtained from CellBank Australia (https://www.cellbankaustralia.com; Cancer Institute NSW, Australia). The cell cultures were maintained < 6 months after re-animation and confirmed to be negative for mycoplasma using the MycoStrip™ test kit. The T47D breast cancer cell line (an estrogen receptor (ER) and progesterone receptor (PR) positive cell line originally obtained from pleural effusion from 54 yr old patient with infiltrating breast cancer (38, 39) generously was provided by Associate Professor T.E. Hickey, Dame Roma Mitchell Cancer Research Laboratories, Adelaide Medical School. The triple negative breast cancer cell line, MDA-MB-231 was generously provided by Prof D Adelson, University of Adelaide.

Ishikawa cells were maintained in DMEM supplemented with 10% FBS, 100 units/ml each of penicillin and streptomycin, and GlutaMAX™ (2 mM). MFE-280 cells were maintained in DMEM/F12 supplemented with 10% FBS, 100 units/ml each penicillin and streptomycin, and 0.005 mg/ml insulin. 24 hr prior to treatment with steroid hormones, the culture medium was changed to phenol red free DMEM/F12 (21041-025; ThermoFisher Scientific, Tullamarine VIC Australia) supplemented with 10% heat-inactivated charcoal-stripped FBS (CS-FBS) prepared as described previously (40) to minimize pseudo-estrogenic effects of FBS and phenol red (41, 42).

### 2.3 Fuling preparation

Capsules of Fuling (trade name Gynoclear) purchased from Metagenics (Queensland Australia), were opened in a sterile environment. The powdered Fuling content was dissolved in the vehicle dimethylsulfoxide (DMSO) to create a series of 2500x stock solutions (0.25 to 7.5 g/ml in DMSO), which in turn were diluted 1:2500 into culture media at final doses for experimental testing. Control groups were untreated and vehicle-treated (0.04% DMSO). Gynoclear capsules contain *Carthamus tinctorius* (Safflower), *Cinnamomum cassia* (Chinese cinnamon), *Poria cocos* (Hoelen), *Paeonia suffriticosa* (Tree peony), *Paeonia lactiflora* (Peony) at 92 mg each and *Salvia miltiorrhiza* (Red sage) at 90 mg, in approximately equal proportions by dry weight (34).

### 2.4 Quantitative real time polymerase chain reaction

Real time quantitative polymerase chain reaction (RT-qPCR) was used to quantify estrogen receptor-α gene *(ESR1)*, estrogen receptor-β gene *(ESR2)* and progesterone receptor gene *(PGR)* transcript levels. Hormone receptor positive breast cancer cell line T47D served as a positive control, whereas *PSMC4* and *IPO8* were used as housekeeping genes for 2ddct standardization (43). Two independent experiments were done with three replicates each. The primers used were *ESR1*= F: GGAATGATGAAAGGTGGGATACG, R: GCATCCAACAAGGCACTGAC, product length = 221bp, annealing temp; 60°C. *ESR2* = F: GCTCGCTTTCCTCAACAGGT, R: TGGGCAAGTATAATGGCTTGCAG, product length=130bp, annealing temp: 60 °C, *PGR* = F: AGGTCTACCCGCCCTATCTC, R: AGTAGTTGTGCTGCCCTTCC, product length = 198, annealing temp: 60 °C, IPO8= F: GGTGGGGTGTGAGGTAATCC, R: ACTGGTTGAGCTCGTTCTCG, product length; 201bp, PSMC4= F: TGGAGGTGCAGGAGGAATACA, R: CTGTGGTAGAGCCCACGATG. Product length = 162bp. Briefly, RNA was extracted from cell lines using RNeasy kit (Qiagen, Cat. 74004) as per manufacturer’s instructions. RNA was eluted in 30 µl of RNase free water. Quantification of total RNA was done by Nanodrop spectrophotometry, and integrity was checked *via* 260/280 and 260/230 wavelength ratios. The integrity of 18S and 28S bands was assessed by electrophoretic separation of RNAs on 2% agarose gel. 1.5 µg RNA was reverse transcribed to cDNA using QuantiTect cDNA conversion kit (Cat. 205331) as per manufacturer’s protocol. Briefly, RNA was heated with gDNA wipe buffer for 3 min at 42°C. After adding RT and RT primer to final volume of 20 µl, the RNA was incubated at 42°C for 15 min and 95°C for 2 min and immediately placed on ice. qPCR was done with the Rotor Gene 3000. Reactions were carried out in final volumes of 10 µl with 5 µl Syber Green (KK4601, Kapa Master Mix), 1 µl (1:100 dilution) cDNA, 1 µl of 10 mM forward and reverse primer mixture and 3 µl RNase free water. The qPCR reaction involved initial denaturation at 95°C for 3 min followed by 40 cycles of 95°C for 3 sec and combined annealing extension at 60°C for 20 s. Analysis was done using the standard 2ddCt method. *IPO8* and *PSMC4* served as reference genes. Reactions were carried out in triplicate in two independent experiments.

### 2.5 Western blot

Protein was extracted from T47D (positive control), MDA-MB-231 (negative control) (44, 45), Ishikawa and MFE-280 cells using RIPA lysis buffer and 1% protease inhibitor. The protein was quantified using Pierce™ 660nm Protein Assay Reagent (22660). Briefly, 50 µg of protein was added to the wells and separated electrophoretically with 10% SDS PAGE and transferred to nitrocellulose membrane (46). The protein was incubated with PGR (1:1000, D8Q2J, cat no. 8757, Cell Signaling, Danvers, MA, USA) at 4°C overnight followed by series of washing with 1x TBS-T. Incubation with secondary antibody goat anti-rabbit IgG (ab216773, 1:5000) IRDye^®^ was done for 1 h. α- tubulin (ab7291, 2µg/ml) was used as loading control on the same membrane. Secondary antibody used for α- tubulin was (ab216772, 0.2µg/ml). Specificity of PR antibody was validated by PR negative breast cancer cell line, MDA-MB-231 (**Supplementary figure S1**).

### 2.6 Cell viability assay

Cell viability was assessed by measuring metabolic activity using established methods (47). Ishikawa cells and MFE-280 were plated in 96 well plates at 10^3^ cells per well in 100 µl of DMEM with 10% FBS. After 24 h growth, media with no drug, with vehicle only, or with Fuling (doses ranging from 100 to 3000 µg/ml) were added. After 24h incubation, 100µl of MTT reagent (M5565 from Merck, diluted 1/10 in sterile phosphate buffered saline) was added to each well. Cells were incubated for 4.5 h (37°C, 5% CO_2_), then the MTT solution was removed and the formazan product was dissolved in 100 µl DMSO per well. Foil covered plates were agitated on a shaker at room temperature for 15 min, then assessed on a plate reader (absorbance 570 nm). Three independent experiments were done with 3 to 4 replicates each.

### 2.7 Wound closure assay

Ishikawa and MFE-280 cells were plated at 3 x 10^4^ cells per well and incubated for 24h to allow growth approaching full confluency prior to wound generation. The circular wounds were created by brief suction with a sterile micropipette tip as per published protocols (48). Wells were rinsed to remove debris; then media with no drug, with the vehicle DMSO only, or with Fuling (80ug/ml) were added and plates were incubated for 24 h. Quantification of percent wound closure was done using Image J (National Institutes of Health; imagej.nih.gov/ij/) and GraphPad Prism (San Diego CA, USA) software to calculate final areas of the wounds at 24 h standardized to the initial areas of the same wounds at 0 h, as per published protocols (48). Three independent experiments were done with 5 replicates each.

### 2.8 Cell invasion assay

Transwell invasion was carried out using 8µm pore filter Boyden chambers (Corning #3422) prepared with 40µl per well of 0.025 mg/ml Matrigel (E1270-10ml, Merck, Lot# 087M4177V) (49). On the experimental day, the Matrigel was rehydrated with 50µl serum-free media, 1 h at 37°C. Cell lines were grown in T75 flasks to approximately 60% confluency, then starved in media with 2% serum for previously optimized periods (30h for Ishikawa cells and 15h for MFE-280). After trypsinization, cell lines were suspended in serum-free media with no drug, vehicle, or Fuling. Cells (2x10^5^ per well) were added to the upper chamber; the lower chamber contained 700ul of media with 10% FBS as a chemoattractant. After 24 h incubation (37 °C, 5% CO_2_), non-invaded cells on the cis side were removed gently with a cotton swab, and remaining invaded cells on the trans side were fixed with 70% ethanol, stained with 0.4% crystal violet, and imaged. Numbers of invaded cells per standardized field of view were counted (with treatment blinded) by two independent researchers and averaged. For the tests of hormonal effects, cell lines were incubated with optimal doses of E2 (1nM for Ishikawa cells and 100 pM for MFE-280), or P4 (100nM) for 48 h. Fuling (80µg/ml) was added at the start of the invasion assay with the inoculation of cells into the upper chamber. ICI182,780 (100nM) and RU486 (1µM) were added 2hr prior to treatments with E2 or P4. Two independent experiments were done with two replicates each.

### 2.9 Spheroid invasion assay

To prepare the well plates, polyhema (P3932-10G) coating solution was made with 0.3g/ml of polyhema crystals dissolved in 95% ethanol, shaken 48 h at 37°C. 96-well round bottom plates (Corning-3799) were coated with 30 µl of polyhema per well in the dark, then exposed to UV light for 2 hrs prior to seeding cells. To produce spheroid cancer cell masses, Ishikawa and MFE-280 cell lines were seeded into wells at a density of 5x10^3^ cells/ml in full DMEM, and the plates were centrifuged at 600 rpm for 3 min, then incubated at 37°C in 5% CO_2_ for 48hrs, and cooled on ice for 15 min. 50 µl of 250mg/ml Matrigel in DMEM was added to each well; plates were centrifuged (600 rpm at 4°C for 5 min) and incubated 1h to allow the Matrigel to set. At time zero, media containing no drug, an equivalent dose of vehicle, or Fuling at 100 µg/ml were added (50). Time-lapse images were taken of the same spheroids at 0, 24, 48, 72 and 96 h using NIS-elements software (Nikon, Tokyo, Japan). Parameters of spheroid area, perimeter, and the numbers of cell clusters (particles) that had disseminated into the surrounding Matrigel environment were quantified using NIH Image J software; rates of change were determined from data plotted as a function of time and fit with simple linear regression. Three independent experiments were done with 8 to 9 replicates each.

### 2.10 Statistics

Distribution normality was tested by the D’Agostino-Pearson test. One-way ANOVA was used to assess significant differences across multiple groups (compared to vehicle control; p<0.05), followed with *post-hoc* comparisons (unpaired T-test for parametric; Mann Whitney for non-parametric; see Figure legends). In box plots, rectangles contain half the data values, whiskers show the full range, and horizontal lines indicate median values. Averaged data (in *x-y* plots) are mean ± standard deviation (SD).

## 3 Results

### 3.1 Expression of estrogen and progesterone receptors in endometrial cancer cell lines

**Figure 1:**
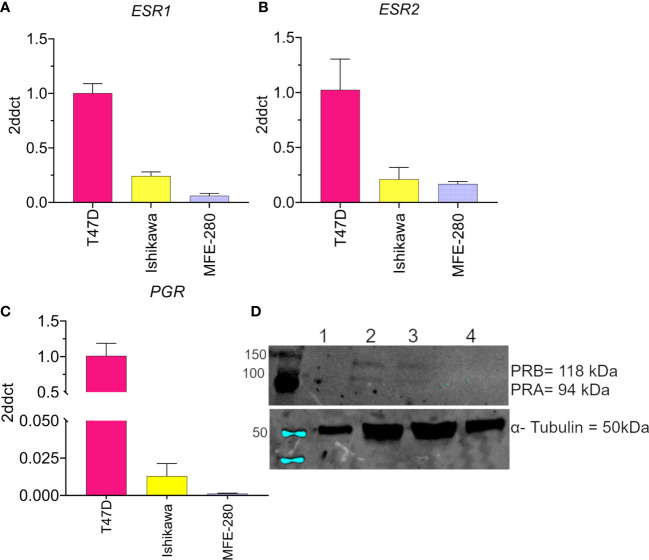
Compiled data confirming the low to moderate expression levels for the hormone receptors **(A)** *ESR1*, **(B)** *ESR2*, and **(C)** *PGR* in Ishikawa and MFE-280 cell lines, and high levels in T47D cells. Transcript levels were analyzed by Livak (2ddct) (76). **(D)** Protein expression of PRA and PRB was evaluated by western blot. Lanes show immunolabeling results for total protein from (1) T47D, 20 µg (2) T47D, 50 µg (3) Ishikawa, 50 µg (4) MFE-280, 50 µg. Upper panel: Neither PR isoform was detected in T47D at 20 µg protein (Lane 1). At 50 µg, both T47D and Ishikawa were positive for PRA (94 kDa) and PRB (118 kDa). MFE-280 cells did not express detectable levels of either PR isoform. Lower panel: Expression of the housekeeping protein α- tubulin (50kDa) in the same samples.

The levels of mRNA transcripts for estrogen receptors (*ESR1* and *ESR2*) and progesterone receptors (*PGR*) were determined in both cell lines using real-time quantitative polymerase chain reaction **Figure 1**. Both EC cell lines showed low expression of *ESR1* (**Figure 1A**) and *ESR2* (**Figure 1B**) as compared to the T47D breast cancer line used as a positive control. However, a relatively higher expression of *ESR2* was found in MFE-280 cells as compared to *ESR1*. This finding is supported by the fact that low grade EC is associated with positive *ESR1* status and better prognoses, while higher *ESR2* expression (hence decreased *ESR1* to *ESR2* ratio) in high grade EC is related to shorter disease-free survival and poor prognosis (51). Expression of *PGR* mRNA (**Figure 1C**) in Ishikawa cell line was confirmed, while, was negligible in MFE-280.

Protein expression of PR isoforms PRA and PRB was assessed by western blot (**Figure 1D**). Protein expression of PRA and PRB was confirmed in Ishikawa cell lines but not detected in MFE-280. The lack of expression of progesterone receptors in high grade EC and its relevance to poor prognosis have been described previously (52). ER and PR loss is an independent prognostic factor for EC (53), and important clinically for differentiating high and low risk groups to guide treatment.

### 3.2 Hormonal regulation of viability and invasiveness in endometrial cancer cell lines

**Figure 2:**
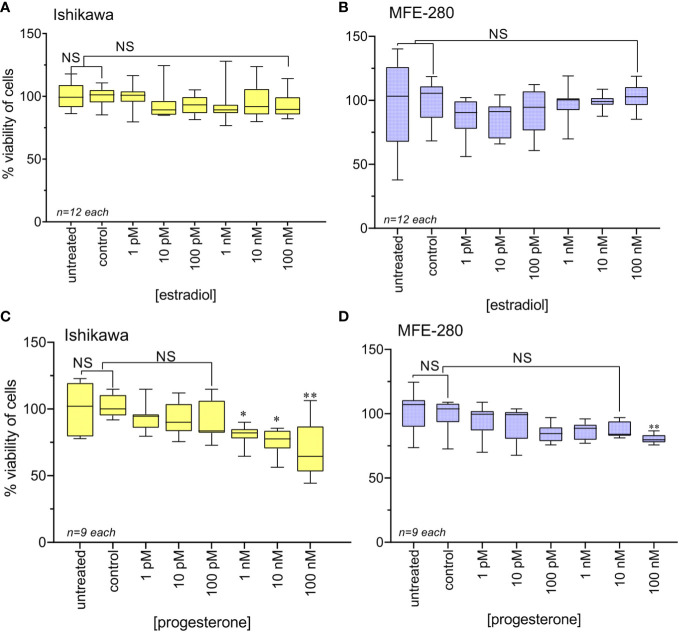
Viability of Ishikawa and MFE-280 cells after treatment with estradiol or progesterone for 48h, as compared with viability of untreated and vehicle control cells. Box plot data summaries are shown for **(A)** Ishikawa and **(B)** MFE-280 cells treated with estradiol, and **(C)** Ishikawa and **(D)** MFE-280 cells treated with progesterone over broad ranges of concentrations. (unpaired T test: *p<0.05; **p<0.01; NS not significant).

Effects of ovarian hormone receptor signaling on the viability of Ishikawa and MFE-280 cells were assessed using the MTT assay, as summarized in **Figure 2**. Treatment with physiological doses of estradiol (1 pM to 100 nM) for 48h caused no change in cell viability in either cell line (**Figures 2A, B**). In agreement with prior work (54), physiological doses of progesterone at 1 nM and above decreased Ishikawa cell viability (**Figure 2C**). In contrast, MFE-280 cells showed reduced viability only at the highest concentration, 100 nM of progesterone (**Figure 2D**). These data provide the first assessment of hormone sensitivity in MFE-280 cells.

**Figure 3:**
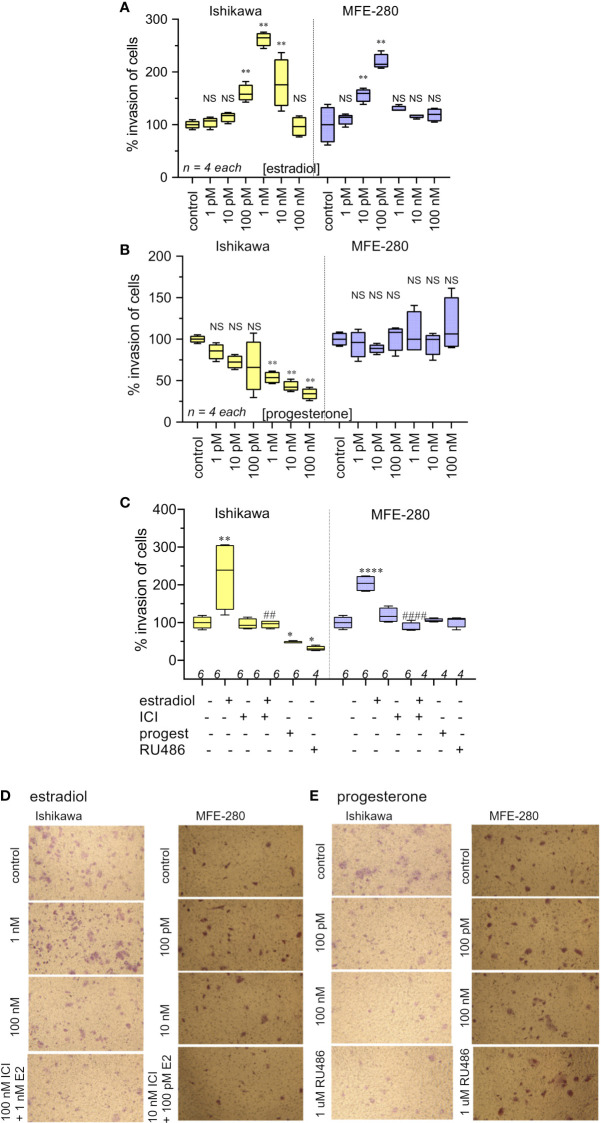
Box plot summary of transwell invasion by Ishikawa and MFE-280 cells in response to treatments with **(A)** estradiol; **(B)** progesterone; **(C)** hormones (1 nM estrogen for Ishikawa and 100 pM for MFE-280, and 100 nM progesterone for both cell lines) with and without receptor antagonists 100 nM ICI 182,870 (estrogen) or 1 µM RU486 (progesterone). Significant differences are indicated by (*) for comparisons with control, or (#) for comparisons with estradiol treatment. (unpaired T test: * or #p < 0.05; ** or ##p < 0.01; **** or ####p < 0.0001; NS not significant). (*n*) values are indicated above the x-axes. **(D, E)** Representative images from transwell invasion assays showing the numbers of Ishikawa and MFE-280 cells that crossed ECM barrier filters in response to serum gradients, in the presence of indicated doses of estradiol **(D)** or progesterone **(E)**, with and without hormone receptor antagonists ICI 182,780 (100 nM) or RU486 (1 µM). Images represent results summarized in **Figure 2**.

Effects of the hormones estradiol and progesterone on invasiveness of Ishikawa and MFE-280 cells were quantified by measuring transwell invasion through an extracellular matrix barrier layer (**Figure 3**). Ishikawa and MFE-280 cells were incubated with a range of concentrations of estradiol and progesterone (1 pM to 100 nM) for 48 h. Data compiled as box plots showed the effects of estradiol on Matrigel invasiveness yielded bell-shaped curves for both Ishikawa and MFE-280 cells. Dose-dependent increases in percent cell invasion in the presence of estradiol showed maximal effects at 1nM for Ishikawa and 100 pM for MFE-280 cells, and reduced responses at higher concentrations (**Figure 3A**).

Progesterone caused a dose-dependent decrease in invasiveness of Ishikawa cells from 1nM-100nM (**Figure 3B**), showing effectiveness at concentrations corresponding to those observed during the menstrual cycle (13). However, progesterone had no effect on the invasiveness of MFE-280 cells (**Figure 3B**), perhaps reflecting a difference in functionality or subtype of the progesterone receptor that is expressed in this cell line (55). Treatment of Ishikawa cells with the progesterone receptor antagonist RU486 appeared to cause a small additional inhibition but had no effect on the progesterone-insensitive MFE-280 cells. The inhibition of invasion by mifepristone in Ishikawa cells is supported by the fact that when Ishikawa cells were treated with 1 µM mifepristone for 12 h, downregulation of genes associated with cell adhesion was observed (56). However, when cells were co-treated with 100 nM progesterone and 1µM RU486, no significant change in invasion was seen as compared to progesterone or RU486 alone. Co-treatment of MFE-280 cells by progesterone and RU486 caused no significant change in invasion compared to control (Supplementary Figure S2).

Estradiol-enhanced invasiveness in both cell lines was reversed by the estrogen receptor (ER) inhibitor ICI182,780 at 100nM (**Figure 3C**), demonstrating that the enhanced response was estrogen receptor mediated. This profile fits with known patterns of estrogen activity described for the female menstrual cycle, during which estrogen exerts maximal effect on endometrium at an upper limit near 50 pM; beyond that concentration the endometrial receptors become desensitized and the magnitude of the estrogenic response declines (13).

Images illustrating the invasion responses (corresponding to data summarized in **Figures 3A–C**) are shown in **Figures 3D, E**. Ishikawa cells showed maximum invasion at 1 nM estradiol, and MFE-280 cells showed maximum invasion at 100 pM. Progesterone caused dose-dependent inhibition of invasion in Ishikawa cells, but had no effect on invasion in MFE-280 cells.

### 3.3 Fuling reduces the invasiveness of Ishikawa and MFE-280 cells through extracellular matrix barriers, and decreases motility

**Figure 4:**
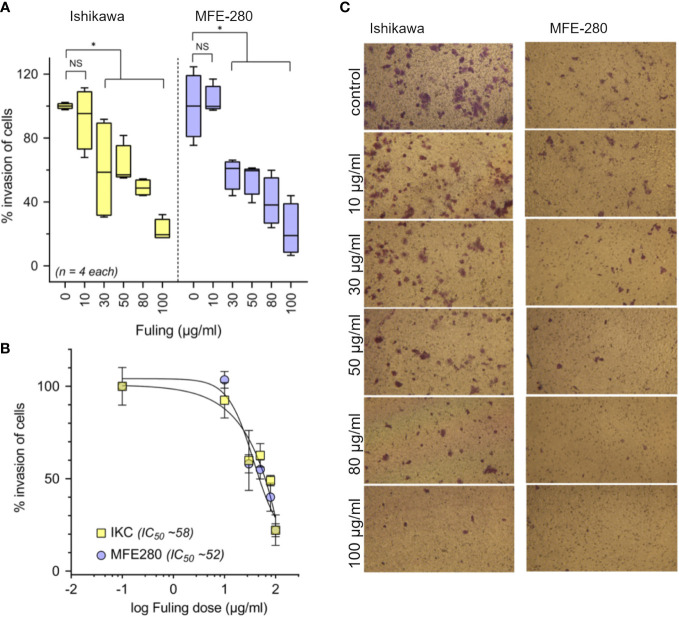
Dose-dependent inhibition of invasion of Ishikawa and MFE-280 cells by Fuling. **(A)** Box plot summaries of compiled data for percent invasion in transwell assays over a range of Fuling doses. (unpaired T-test: *p < 0.05 *vs* control; NS not significant). **(B)** Dose response curves were used to determine of the doses of Fuling that caused half-maximal inhibition of invasion (IC_50_) values, which were 52 and 58 µg/ml in Ishikawa and MFE-280, respectively. **(C)** Representative images Ishikawa and MFE-280 cells, showing the inhibitory effects of Fuling on transwell migration.

Treatment with Fuling extract caused dose-dependent inhibition of the invasiveness of Ishikawa and MFE-280 cells, as measured by transwell assays quantifying percent cell migration through an extracellular matrix barrier (Matrigel) at 24 h (**Figure 4**). Box plot histograms show Fuling reduced invasiveness in both cell lines (**Figure 4A**), yielding 50% inhibition at comparable Fuling doses of 58 µg/ml for Ishikawa and 52 µg/ml for MFE-280 cell lines (**Figure 4B**). Representative images of invasion results for each treatment are illustrated in **Figure 4C**.

**Figure 5:**
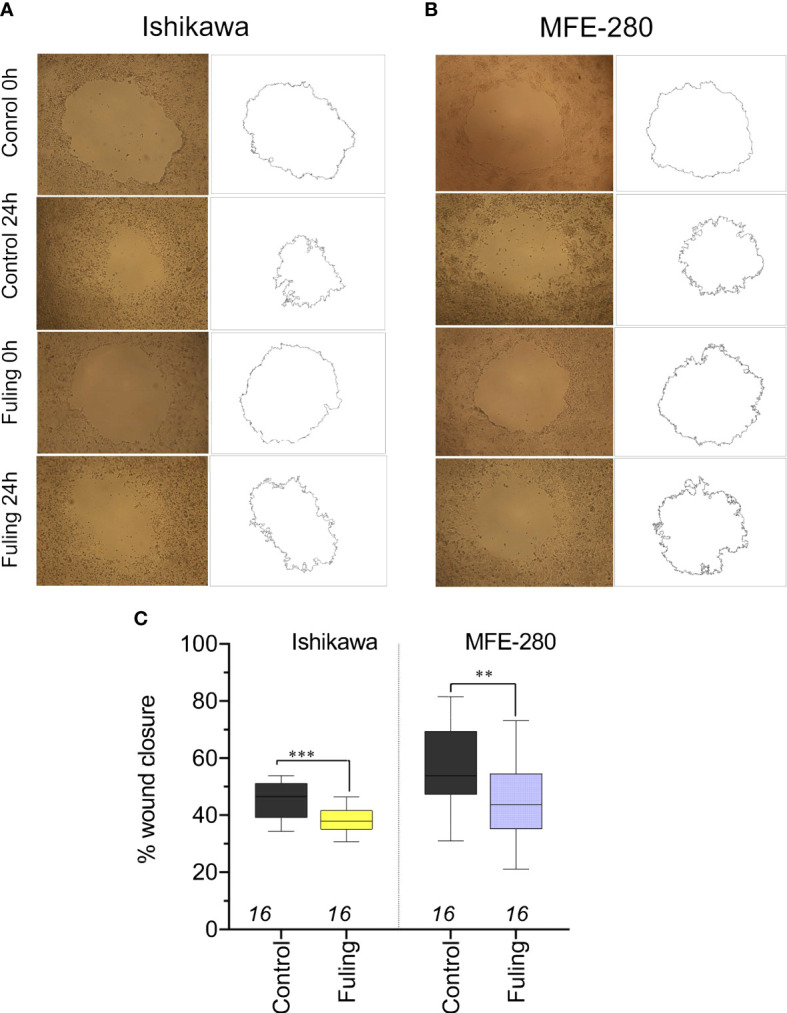
Decreased motility measured by reduced wound closure rates in plated Ishikawa and MFE-280 in cultures treated with 80 µg/ml Fuling, as compared with vehicle control (images at 0 and 24 h). **(A, B)** Representative images and corresponding outlines of the initial wounds and the final wound sizes after incubation with vehicle (0.04% DMSO) or Fuling (24 h). **(C)** Box plot summary of compiled data for percent wound closure, showing significant block by Fuling in both cell lines (unpaired T-test: **p < 0.01; ***p < 0.001).

To determine whether Fuling also inhibited the rates of two-dimensional surface migration in both cell lines, circular wounds were made in plates of confluent cells, and the rates of wound closure were quantified by NIH-Image software analysis, after incubation with 80 µg/ml of Fuling or vehicle (DMSO) for 24 h. Fuling significantly inhibited migration, slowing the closure of wounds in both cell lines as compared to the control (vehicle-treated) group (**Figure 5**). Images illustrating the wound closure are shown in **Figure 5A, B**. Percent wound closure (**Figure 5C**) was calculated by standardizing the wound area measured after 24 h incubation to the initial area for the same wound measured immediately after wound formation (see Methods for details).

### 3.4 Fuling overrides the estradiol-mediated potentiation of invasiveness

**Figure 6:**
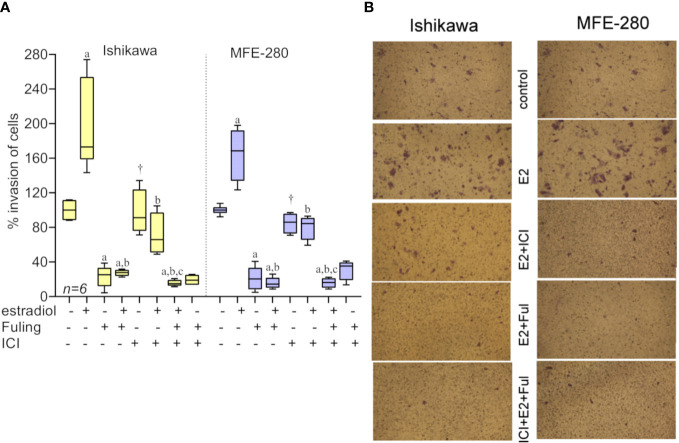
Transwell invasiveness of Ishikawa and MFE-280 cells at 48 h after treatments with estradiol (at 1 nM for Ishikawa, and 100 pM for MFE-280), with and without with Fuling (80µg/ml), ER antagonist ICI182,780 (100 nM), or both inhibitors combined. **(A)** Box plot summary of percent invasion responses in the matrix of treatments indicated below the x-axis. Results of multiple statistical comparisons (unpaired T-test) are coded with lower case letters: ‘a’ indicates statistical significance as compared with control; ‘b’ compared to estradiol alone; and ‘c’ compared to ICI +estradiol; all at p < 0.05. The symbol (†) indicates no significant difference from control. **(B)** Representative images showing stimulatory effects of estradiol on transwell invasion, the strong inhibitory effect of Fuling, and the moderate impairment of invasion by ICI182,780.

To assess the effects of Fuling on estradiol-mediated increases in invasiveness, both cell lines were incubated with estradiol for 48h in the presence and absence of Fuling. Fuling blocked invasiveness uniformly, overriding the potentiating effects of estradiol on invasion (**Figure 6**). Treating cells with the estrogen receptor antagonist ICI182,780 (100 nM; for two hours before treatment with estradiol) did not alter the blocking effect of Fuling, indicating that Fuling inhibits motility of endometrial cancer cells independently of estrogen receptor antagonist. However, possible interactions of Fuling with ER-α, ER-β and the newly described G- protein coupled estrogen receptor GPER (57, 58) remain unexplored and offer an intriguing area for further work.

**Figure 7:**
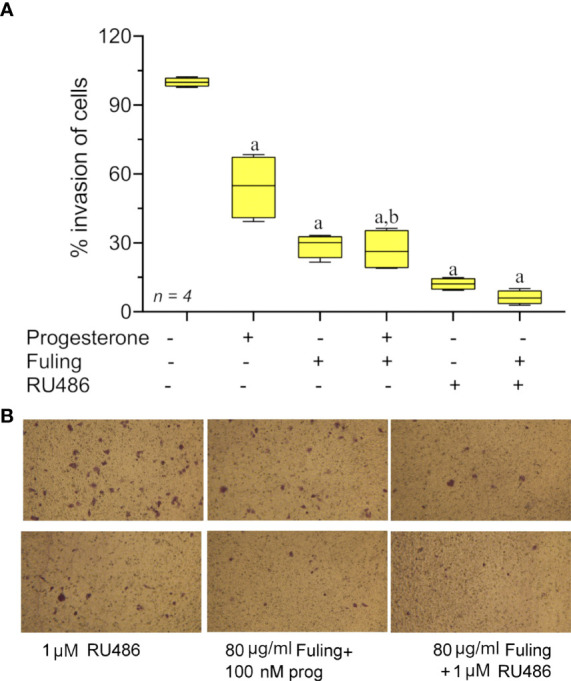
Transwell invasion responses in Ishikawa cells treated with 100 nM progesterone and 80 µg/ml Fuling. **(A)** Box plot summary of percent invasion (at 24 h). (unpaired T-test: ‘a’ is significant *vs* control, ‘b’ is significant *vs* progesterone; p < 0.05) **(B)** Representative images showing the effects of Fuling on Ishikawa cell transwell invasion with and without progesterone co-treatment.

Progesterone alone blocked Ishikawa cell invasion (as described above in **Figure 3**). The effect of Fuling applied in combination with progesterone on the inhibition of invasion was tested by pretreating cells with 100 nM progesterone for 48h prior to transwell invasion assays done with and without Fuling (**Figure 7**). Fuling alone was more effective than progesterone alone in inhibiting Ishikawa cell invasion (**Figure 7A**), based on the numbers of transwell-migrated cells (**Figure 7B**). The level of block generated by Fuling showed no additive effects of progesterone; the effect of combined Fuling and progesterone was not different than treatment with Fuling alone in suppressing motility. The progesterone receptor antagonist RU486 similarly did not add any significant effect when combined with Fuling, suggesting Fuling might be acting at pathways closer to the direct mechanisms of cell motility, and downstream of the hormone receptor signaling.

### 3.5 Fuling reduces the dispersal of Ishikawa cells from spheroid tumor masses

**Figure 8:**
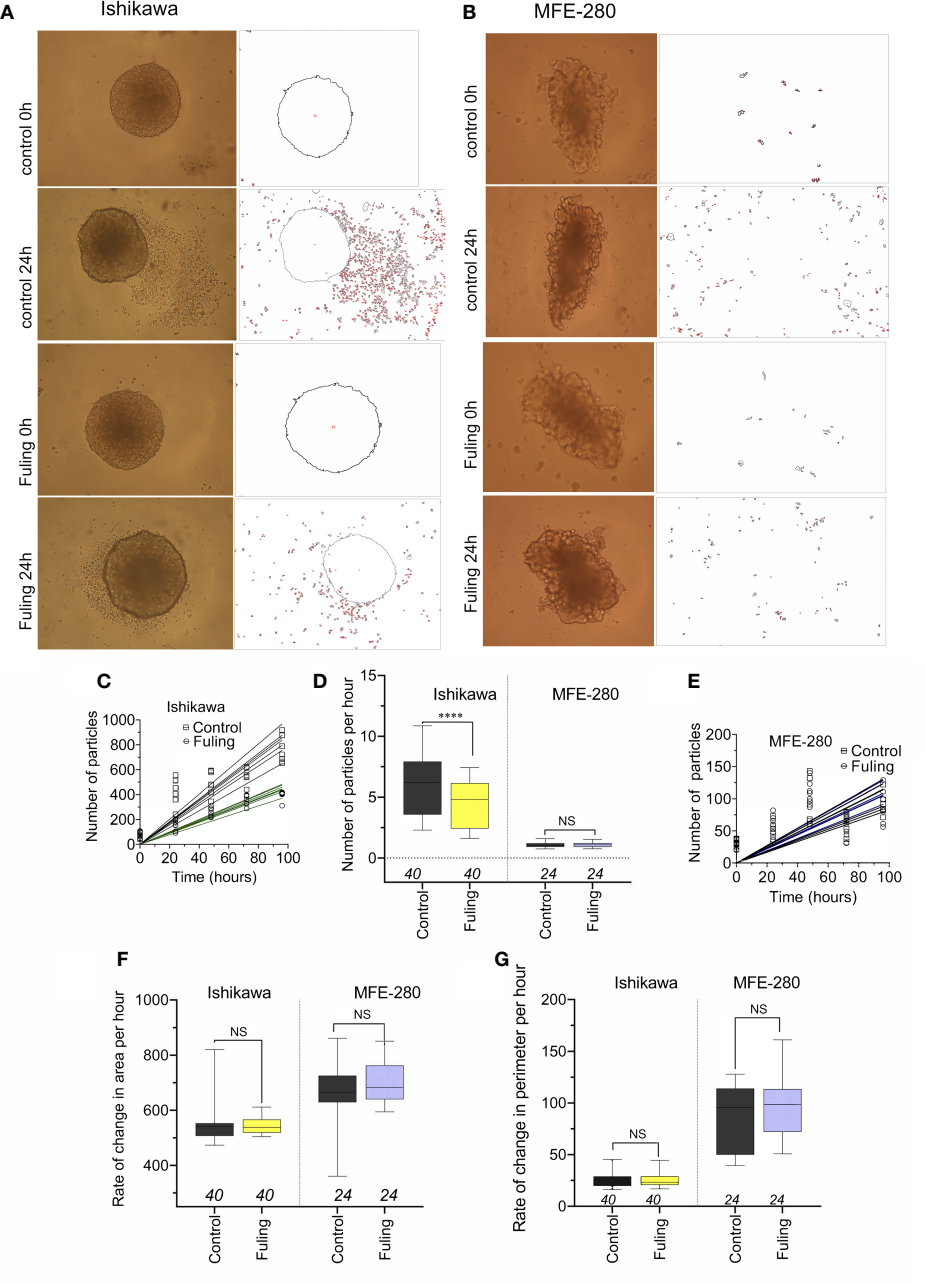
Dispersal of cancer cells from solid tumor mass spheroids of Ishikawa and MFE-280 cells, with and without treatment with 100 µg/ml Fuling. **(A, B)** Representative images showing outlines of spheroids, surrounded by cancer cell clusters (particles) that disseminated into the surrounding matrigel, detected by NIH ImageJ at 0 and 96h of treatment with Fuling or vehicle control. **(C, D, E)** Simple linear regressions of numbers of particles around spheroids of both cell lines **(C,E)**, and a box plot summary of Ishikawa dispersal rates **(D)** as new particles per hour. **(F,G)** Box plots of rates of change in **(F)** spheroid area (µm^2^/h) and **(G)** spheroid perimeter (µm/h) showed no differences in growth rates, with or without Fuling treatment in either cell line. Significant differences were analyzed by Mann Whitney test: ****p < 0.0001; ns not significant).

Solid tumor spheroid masses were generated for Ishikawa and MFE-280 cancer cell lines, embedded in an extracellular Matrigel environment to evaluate rates of cell cluster dispersal as a model for tissue invasiveness. When the spheroids developed from Ishikawa cells were incubated with 100 µg/ml of Fuling for 96h, the rate of dispersal of cancer cell clusters into the surrounding zones was significantly reduced as compared to the control group (**Figure 8**). Images in **Figure 8A** illustrate the robust dispersal of Ishikawa cells, which was blocked by Fuling. In contrast MFE-280 cells showed relatively low rates of dissemination from the core tumor under the conditions tested (**Figure 8B**). Time lapse series of images allowed assessment of the rates of appearance of new Ishikawa cell clusters (particles) as a function of time in culture, and showed distinctly reduced rates in Fuling treatment (**Figure 8C**), quantified in box plots of rates of appearance per hour (**Figure 8D**). MFE-280 showed much lower numbers (note the smaller y-axis scale) and no discernable effect of Fuling (**Figure 8E**).

There were no differences in the rates of increase in area or perimeter of the spheroids over 0 to 96h, comparing the control and Fuling treatments (**Figures 8F, G**). The equivalent growth confirmed that viability of the tumor cells was not impaired, and that while Fuling did strongly block motility, this was not an indirect result of compromised cell viability.

### 3.6 Doses of Fuling that are effective in controlling invasiveness are not toxic

**Figure 9:**
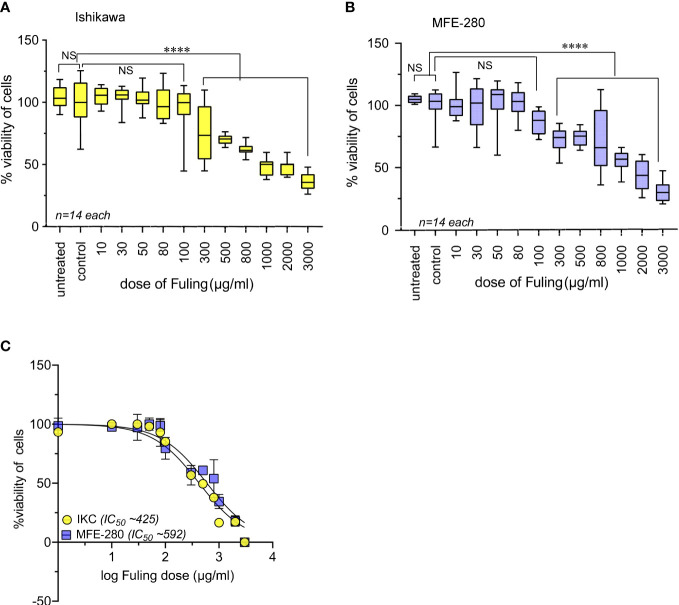
High doses of Fuling reduced cell viability in both cell lines. Box plots summarize percent cell viability standardized to vehicle control for **(A)** Ishikawa and **(B)** MFE-280 cells as a function of Fuling dose. (unpaired T-test: ****p < 0.0001 versus control; NS is not significant). **(C)** Log dose-response curves yielded values for doses of Fuling that caused half-maximal loss of viability, at approximately 425 µg/ml in Ishikawa (IKC) and 592 µg/ml in MFE-280 cell lines.

Effects of Fuling on cell viability at 24 h of treatment were assessed using doses ranging from 10 to 3000 µg/ml (**Figure 9**). High doses of Fuling compromised cell viability (at 500 µg/ml to 3000 µg/ml) in both Ishikawa (**Figure 9A**) and MFE-280 (**Figure 9B**) cell lines. In contrast, Fuling was not toxic in the low to moderate range of doses (10 µg/ml to 100 µg/ml) which were found (see **Figure 4** above) to be effective for inhibiting invasion. Dose-response curves plotted on a log scale (**Figure 9C**) showed that the doses of Fuling which imposed half-maximal decreases in viability were 7- to 11-fold higher than those needed to half-maximally block invasiveness in both cell lines.

## 4 Discussion

Endometrial cancer is a clinical issue that despite its high occurrence remains inadequately treated and seriously impacts quality of life for many women. Outcomes here support the novel proposal that Fuling might be a valuable adjunct that could be used to control endometrial cancer by stopping cancerous cells from invading surrounding tissue at low (non-toxic) doses. The effective doses for preventing cancer cell invasiveness are comparable to those being taken by humans as medicinal supplements. Preventing cancer cell spread could enhance the success of parallel standard clinical treatments which are aimed at removing primary tumors.

Current treatment options for endometrial cancer are limited. Chemotherapy and radiotherapy target rapidly dividing cells (59), but cause adverse effects such as intractable vomiting, diarrhea, immunosuppression and hair loss (60–64). Interest has been escalating in Traditional Chinese Medicines which are thought to work *via* synergistic combinations of pharmacological agents that appear to improve outcomes in disease conditions including cancers (26, 65), driving consumer trends towards complementary and alternative therapies (66). Ongoing research is defining possible mechanisms of action. Common molecular and genetic features between endometriosis and cancer (35, 37) prompted our hypothesis that Fuling might have beneficial effects in endometrial cancer, an idea that has not to our knowledge been tested previously. Work here demonstrated the effects of Fuling in reducing invasiveness of two endometrial cancer cell lines, categorized as FIGO grades I and III (MFE-280). The Ishikawa cell line (67) is thought to be a model for progestin-responsive types of EC (68). The MFE-280 cell line is more representative of recurrent and poorly differentiated endometrial cancers which are resistant to hormonal therapy (69).

The major outcome of this study was the demonstration that Fuling inhibits invasiveness in both high and low grades of EC cells, and that this effect was achieved at doses estimated to be comparable to those being used in clinical testing and traditional practice. In an ongoing clinical trial (33, 34), patients diagnosed with endometriosis are being treated with Fuling at an estimated final *in vivo* dose of ~70 µg/ml Fuling, which is within the same range as the IC_50_ values defined here (i.e., 52 and 58 µg/ml Fuling were effective in blocking invasiveness in two grades of EC cell lines (see **Figure 4**). Results of the clinical trial will be of substantial interest when available.

The second important outcome of this work was that doses of Fuling needed for controlling invasive spread were not cytotoxic, suggesting Fuling (already approved for human use) has potential as an adjunct therapy which could restrain EC invasive spread prior to and during clinical interventions aimed at eradicating primary tumors. If successful, using Fuling as an adjunct therapy would be predicted to reduce rates of cancer cell escape and EC recurrence. Prior *in vivo* studies of Fuling have been conducted with animal models of endometriosis, but given the high doses of Fuling typically administered, the outcomes of interest were focused primarily on cell death (30), and might not correspond with physiological mechanisms that underlie the proposed health benefits in humans.

Results here indicate the mechanism by which Fuling blocks invasiveness appears to be independent of the estrogen and progesterone pathways. Fuling consistently inhibited invasion in all conditions, overriding the potentiating effects of estradiol in both cell lines. The estrogen receptor antagonist ICI 182,780 added a small but discernable level of inhibition to the effect of Fuling, suggesting an additive effect. Progesterone (100 nM) inhibited invasiveness in Ishikawa cells as expected, but the magnitude of inhibition of invasion by Fuling was greater, and not reversed by co-application of the progesterone receptor inhibitor RU486. The MFE-280 cell line used in our study did not respond to progesterone treatment, supporting other work showing that progesterone receptors are downregulated or develop resistance progressively with increasing grades of EC and poorer prognoses (51, 52, 70, 71). Work here showed the MFE-280 invasiveness was blocked by Fuling, highlighting this agent as a candidate for new strategies to address high grade, poorly differentiated endometrial tumors that do not respond to conventional hormonal therapies. Fuling in other studies has been shown to influence multiple intracellular pathways in different cancer types (**Table 1**), including downregulation of MMP-9, TGF-β and upregulation of tumor suppressor genes. The intracellular pathways affected by the low doses used here and their links to motility control remain to be defined.

Fuling merits exploration as an adjunct treatment in combination with other EC therapeutics (72). For example, ICI182,780 (fulvestrant) is an established FDA-approved drug for clinical use in hormone receptor-positive metastatic breast cancer (73), but few trials have explored the efficacy of ICI182,780 for treating endometrial cancer. In a Phase I trial for EC, the drug was found to be well tolerated and associated with complete remission of estrogen-induced endometrial growth (74). However in Phase II, variability arose, with only 0-3% of patients showing a complete response (defined as lesion disappearance) (75, 76); 11.4-13% patients showing a partial response (50% reduction in tumor size); and 29% continuing to show stable disease (maintaining the original tumor size) (75). Based on the broad range of effects that have been described for traditional herbal medicines, Fuling could be tested for effects in enhancing efficacy or reducing doses needed for agents such as ICI182,780, for counteracting drug resistance, or for supportive care in women with cancers such as ovarian cancer (26).

Medroxyprogesterone acetate has to date been the standard therapy to treat metastatic or inoperable endometrial cancers, and to preserve fertility; however, success rates can be low (15-25%) with high rates of recurrence, due in part to acquired resistance to progesterone therapy (18, 77, 78). The paucity of progesterone receptors in high grade or metastatic endometrial cancers effectively eliminates this treatment option for patients with advanced cancer (52). Meta-analysis of six randomized controlled trials showed hormone therapy did not improve survival in patients with advanced or recurrent endometrial cancer (70). The possibility that Fuling might be able to control these resistant advanced cancers is an exciting prospect.

## Data availability statement

The raw data supporting the conclusions of this article will be made available by the authors, without undue reservation.

## Author contributions

Conceptualization and methodology, SK, AV. Investigation, SK, AV. Writing—original draft preparation, SK, AY. Writing—review and editing, AY, CR. Supervision, AY, CR. Funding acquisition, AY. All authors contributed to the article and approved the submitted version.
