# Supplementary material for: Invasiveness of endometrial cancer cell lines is potentiated by estradiol and blocked by a traditional medicine Guizhi Fuling at clinically relevant doses
Source: Front Oncol. 2023 Jan 16;12:1015708. doi: 10.3389/fonc.2022.1015708 (PMC9885141; doi:10.3389/fonc.2022.1015708)

Supplementary Materials

Supplementary Figure S1. Specificity of anti-PR antibody was validated using the PR-negative breast cancer cell line, MDA-MB-231. (A) Nitrocellulose membrane loaded with samples of MDA-MB-231 protein (1) 20 µg, (2) 40 µg, (3) 50 µg. No protein expression of either isoform (PRA & PRB) was detected in MDA-MB-231. (B) Bands correspond to housekeeping protein α- tubulin (50 kDa) in the same samples as (A). Uncropped full membrane views of results in Figure 1D, showing (C) PR expression in both cell lines; and (D) α- tubulin in the same samples). Lanes were: (1) T47D, 20 µg; (2) T47D, 50 µg; (3) Ishikawa, 50 µg; (4) MFE-280, 50 µg.

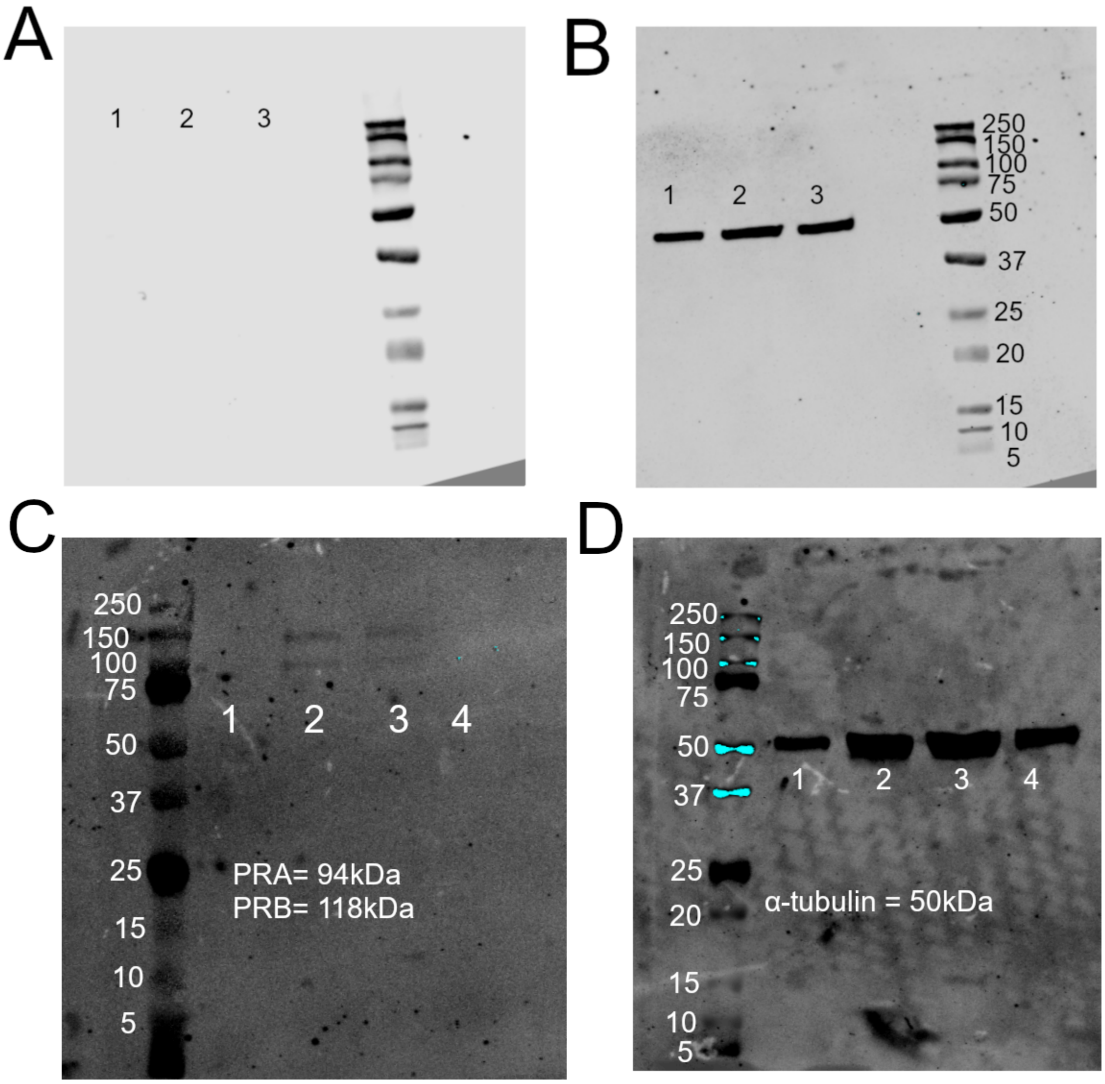

Supplementary Figure S2. Box plot summary of transwell invasion of Ishikawa and MFE-280 cells in response to co-treatment with 100 nM progesterone and 1 µM RU486. No significant changes in invasion were seen for either of the cell lines after combined treatment, as compared with treatments with either progesterone or RU486 alone. One-way ANOVA was used to assess significant differences across groups compared to vehicle control (* p<0.05, followed with post-hoc comparisons (unpaired T-test). Data are compiled from two independent experiments.

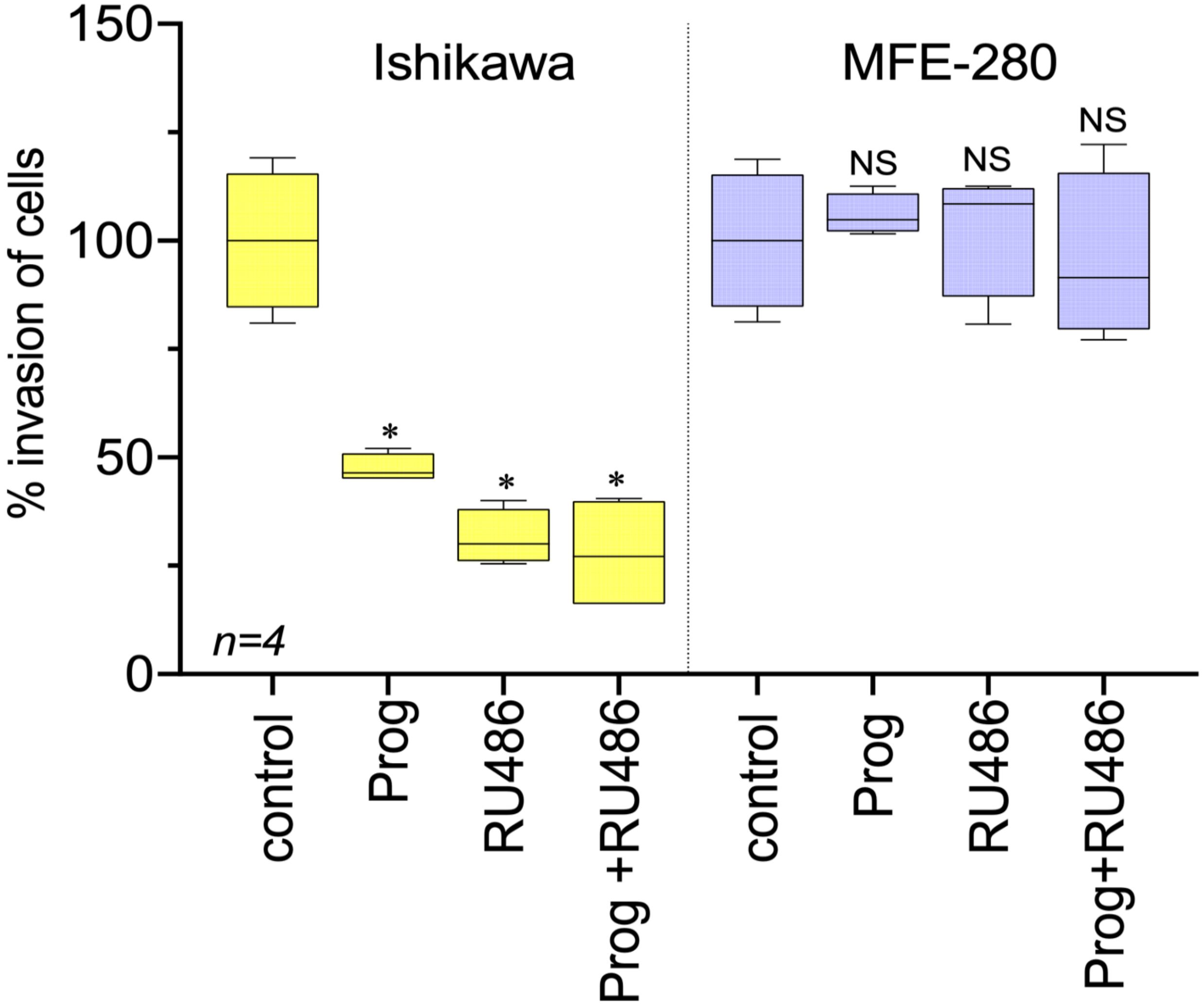

Supplement: Supplementary file 1 [file DataSheet_1.pdf]
